# Induction-Phase Peripheral Perfusion Dynamics and Rocuronium Neuromuscular Blockade Onset: A Retrospective Cohort Study

**DOI:** 10.3390/jcm15103601

**Published:** 2026-05-08

**Authors:** Su Yeon Cho, Dong Joon Kim, Ki Tae Jung

**Affiliations:** 1 Department of Anesthesiology and Pain Medicine, Chosun University Hospital, College of Medicine, Chosun University, Gwangju 61453, Republic of Korea; isycho@chosun.ac.kr (S.Y.C.); djkim@chosun.ac.kr (D.J.K.); 2 Department of Medicine, Graduate School, Chosun University, Gwangju 61452, Republic of Korea; 3 Medical Research Institute, Chosun University, Gwangju 61452, Republic of Korea

**Keywords:** neuromuscular blockade, rocuronium, perfusion index, photoplethysmography, anesthesia induction, hemodynamics

## Abstract

**Background:** Rocuronium onset time shows interindividual variability, yet its hemodynamic determinants remain incompletely characterized. The peripheral perfusion index (PI), derived non-invasively from pulse oximetry, reflects integrated cardiac output and peripheral vascular tone. We hypothesized that early PI dynamics during anesthesia induction are associated with rocuronium onset time. **Methods:** This single-center retrospective observational cohort study included 1377 adults who received rocuronium 0.6 mg/kg under a standardized induction protocol with quantitative electromyographic train-of-four monitoring. Baseline PI was categorized as low-PI (PI < 0.7), intermediate-PI (0.7 ≤ PI < 1.4), or normal-PI (PI ≥ 1.4), and patients were further stratified by anesthetic method (TIVA or volatile anesthesia), yielding six subgroups. The 60 s PI change after propofol bolus (ΔPI_60_) was selected as the primary dynamic variable and classified into equal-frequency tertiles (Slow-Rise, Mid-Rise, Fast-Rise). Sequential multivariable linear regression model was used to quantify independent contributions of induction-phase variables on onset time. **Results:** In the overall cohort, onset time differed significantly across baseline PI groups (*p* = 0.033), though this was not replicated within either anesthetic subgroup (TIVA: *p* = 0.200; volatile: *p* = 0.137). In contrast, ΔPI_60_ tertile was significantly associated with onset time in both subgroups (both *p* < 0.001), with median onset times of 211, 183, and 170 s in the Slow-Rise, Mid-Rise, and Fast-Rise groups, respectively. In the regression model, ΔPI_60_ tertile produced the largest single increment in model fit (adjusted R^2^ Δ0.060, *p* < 0.001); Slow-Rise and Mid-Rise groups had 32.7% and 18.8% longer onset times relative to Fast-Rise, respectively. **Conclusions:** ΔPI_60_ was most strongly associated with rocuronium onset time among the variables examined, independent of baseline characteristics and anesthetic method. These findings provide hypothesis-generating evidence that induction-phase PI dynamics may serve as a physiologically grounded, non-invasive marker of rocuronium delivery conditions, warranting prospective validation.

## 1. Introduction

During general anesthesia, neuromuscular blocking agent (NMBA) is essential for airway management and surgical muscle relaxation. Current guidelines recommend quantitative neuromuscular transmission (NMT) monitoring whenever NMBAs are administered [1,2]. Rocuronium is widely preferred for its comparatively rapid onset among nondepolarizing agents; however, clinically significant interindividual variability persists [3]. The onset of nondepolarizing NMBAs depends on its transport to the neuromuscular junction (NMJ), which is governed by cardiac output and regional muscle blood flow [3,4]. Pharmacologic modulation of hemodynamics further supports this concept. Increasing cardiac output with ephedrine accelerates the onset of rocuronium by approximately 22–26% [5], whereas reducing cardiac output with esmolol delays its onset [6]. Notably, phenylephrine, despite maintaining arterial pressure without direct inotropic effects, also prolongs onset [7], suggesting that microvascular perfusion and peripheral vascular resistance play a critical role in drug delivery to the neuromuscular junction.

The peripheral perfusion index (PI) is a non-invasive parameter derived from the pulse oximetry photoplethysmographic signal, representing the ratio of the pulsatile to non-pulsatile infrared absorbance at the measurement site [8]. Although PI is measured at the fingertip, its value is determined by systemic hemodynamic factors, including cardiac output, stroke volume, and peripheral vascular tone [8], and thus reflects the integrated systemic hemodynamic state [8]. Accordingly, PI is positioned closer to the peripheral drug delivery mechanism than conventional vital signs, such as heart rate or blood pressure alone [8,9]. Importantly, PI is derived automatically from the pulse oximeter already in routine use during anesthesia, requiring no dedicated setup or calibration beyond standard monitoring [8]. This practical simplicity of PI makes it immediately accessible at the bedside in any clinical setting, and may offer a complementary, real-time hemodynamic indicator alongside mandatory NMT monitoring. During general anesthesia, changes in PI have been shown to correlate closely with changes in cardiac output and stroke volume [10], supporting its use as a dynamic hemodynamic surrogate at the bedside. Since cardiac output is an established primary determinant of NMBA onset time [4], we hypothesized that induction-phase changes in PI may serve as a non-invasive surrogate of the hemodynamic conditions governing rocuronium delivery to the NMJ, and that variations in PI may be associated with interindividual differences in the speed of neuromuscular blockade (NMB) onset.

To date, however, the relationship between PI and rocuronium onset time has not been investigated. We therefore conducted a single-center retrospective observational cohort study in patients who received rocuronium under a standardized institutional anesthesia protocol with concurrent quantitative train-of-four (TOF) monitoring. The aim of this study was to determine whether baseline PI and induction-phase PI dynamics (early changes during induction period) are associated with variability in rocuronium onset time.

## 2. Materials and Methods

### 2.1. Study Design, Setting, and Ethics

This was a single-center retrospective observational cohort study conducted at Chosun University Hospital, Gwangju, Republic of Korea. This study was approved by the Institutional Review Board of Chosun University Hospital (CHOSUN IRB 2025-06-026) and conducted in accordance with the principles of the Declaration of Helsinki (2024 revision) [11]. We used de-identified data from an institutional anesthesia registry derived from VitalDB, which includes high-resolution biosignals collected during routine anesthesia using Vitalrecorder (version 1.18) [12]. The registry was previously approved by the Institutional Review Board (CHOSUN IRB 2024-02-002). The requirement for informed consent was waived owing to the retrospective design.

### 2.2. Data Source

Patient data were extracted from the institutional electronic medical records (EMR) and the anesthesia information management system. The dataset was derived from an institutional anesthesia registry based on VitalDB. From this registry, physiological waveforms and numeric parameters, including PI and NMT monitoring data, were extracted at a 1 s resolution for analysis and calculated according to the measurement variables. All included patients were monitored using GE CARESCAPE B850 (Bx50; GE Healthcare, Chicago, IL, USA) monitors and Masimo Radical-7 pulse oximeters (Masimo Corp., Irvine, CA, USA). Data collected between February 2024 and June 2025 were included in the analysis. All variables used in the analysis were derived from these 1 s resolution recordings and sampled at intervals specified in the following Description of the Variables Section.

### 2.3. Inclusion and Exclusion Criteria

Inclusion criteria were: (1) adult patients aged ≥ 18 years; (2) general anesthesia performed in accordance with the institutional standardized induction protocol, as confirmed by EMR review; (3) rocuronium 0.6 mg/kg as the sole NMBA at induction; (4) pre-induction PI available from the Masimo Radical-7; (5) quantitative NMT monitoring at the adductor pollicis using electromyography (EMG); (6) all vital signs, PI and NMT monitoring data were recorded electronically using Vitalrecorder.

Exclusion criteria were: (1) age < 18 years; (2) use of NMBA other than rocuronium; (3) missing or unreliable PI data; (4) missing or unreliable NMT monitoring data; (5) NMT monitoring not meeting Good Clinical Research Practice (GCRP) standards (e.g., inadequate baseline stabilization, lack of supramaximal stimulation, or signal instability) [13]; (6) onset time > 600 s, considered an extreme outlier likely reflecting measurement or recording artifact or potential dosing inconsistency; and (7) administration of vasopressors during the induction period.

### 2.4. Institutional Standardized Anesthesia Protocol

To ensure clinical homogeneity, only cases adhering to the institutional standardized induction protocol were included. The protocol comprised: (1) preoxygenation with 100% oxygen for 3 min; (2) induction with propofol using bolus administration (2.0 mg/kg in patients < 65 years and 1.5 mg/kg in patients ≥65 years) followed by volatile anesthesia maintenance, or total intravenous anesthesia (TIVA) with target-controlled infusion (TCI) at an effect-site concentration (Ce) of 3.0–4.0 µg/mL using Marsh model; (3) remifentanil TCI at an Ce of 1.5–2.0 ng/mL using Minto model; (4) appropriate calibration, confirmation of supramaximal stimulation, adequate signal stabilization, and confirmation of a stable baseline T1 response over at least two consecutive TOF measurements prior to rocuronium administration; (5) quantitative TOF monitoring was performed by EMG at 10 s intervals until endotracheal intubation; (6) rocuronium 0.6 mg/kg administered as an intravenous bolus after a stable baseline T1 response was confirmed over at least two consecutive TOF measurements; (7) PI was measured continuously via the Masimo Radical-7 pulse oximeter probe placed on the second or fourth finger of the hand contralateral to NMT monitoring and non-invasive blood pressure measurement; (8) The operating room was maintained at a standard ambient temperature (21–23 °C) and patients underwent active prewarming for approximately 5–10 min using a forced-air warming blanket before anesthetic induction; and (9) Rocuronium was stored under refrigerated conditions (2–8 °C) in accordance with manufacturer’s recommendations, and brought to room temperature prior to administration. Cases not meeting these standards were excluded from the analysis.

### 2.5. Description of the Variables

TOF count and T1 height relative to the pre-drug baseline were retrospectively extracted. Onset time, the primary outcome of this study, was defined as recommended by the GCRP, as the time from the end of rocuronium injection to 95% suppression of T1 relative to baseline [13].

Baseline PI was defined as the mean PI value over the 2 min window before induction of anesthesia. PI values were extracted at 15 s intervals following propofol bolus administration: t0 (immediately after propofol bolus), t15, t30, t45, and t60 (60 s after propofol bolus). ΔPI was defined as the change in PI from t0 to a given time point after injection, expressed as a percentage:

ΔPI(t) (%) = [[PI(t) − PI(t0)]/PI(t0)] × 100.

Vital signs, including heart rate (HR), non-invasive blood pressure, and oxygen saturation, were measured at 3 min intervals. Baseline mean blood pressure (MBP) and baseline HR were defined as the values recorded before propofol administration. ΔHR and ΔMBP were defined as the change from the baseline value to the value recorded at the onset, respectively.

Other variables, such as age, sex, height, weight, and body mass index, were obtained from the EMR. American Society of Anesthesiologists Physical Status (ASA-PS) class, presence of hypertension, and presence of diabetes mellitus were confirmed from the preoperative assessment records. Core body temperature values using esophageal thermometer immediately after endotracheal intubation were used.

### 2.6. Study Groups

Based on the distribution of PI in healthy adults reported by Lima et al. [9], in which the population median was 1.4 (interquartile range, IQR 0.7–3.0), baseline PI was categorized into three groups: low-PI (PI < 0.7), intermediate-PI (0.7 ≤ PI < 1.4), and normal-PI (PI ≥ 1.4). Within each PI group, patients were further stratified by anesthetic method: the volatile anesthesia group and the TIVA group.

For the secondary analysis, patients were additionally classified into three equal-frequency tertiles based on ΔPI at the optimal time point, determined by evaluating Spearman rank correlation with onset time and AUC at each available interval following propofol administration (t15, t30, t45, and t60). The tertile cutoffs were derived from the overall distribution of ΔPI at the selected time point, yielding three groups: Slow-Rise, Mid-Rise, and Fast-Rise. Within each ΔPI tertile, patients were similarly stratified by anesthetic method.

### 2.7. Outcomes

The primary outcome was rocuronium onset time compared across baseline PI groups (low-PI, intermediate-PI, and normal-PI) in the overall cohort and separately within each anesthetic subgroup (TIVA and volatile anesthesia), given the known influence of anesthetic method on peripheral hemodynamics and rocuronium pharmacodynamics. The secondary outcome was rocuronium onset time compared across ΔPI tertiles (Slow-Rise, Mid-Rise, and Fast-Rise).

### 2.8. Sample Size

An a priori sample size calculation was performed for the primary outcome, rocuronium onset time, compared across three baseline PI groups. Based on prior studies showing that hemodynamic modulation may alter rocuronium onset time by approximately 20–26% [5,6,7], a clinically significant difference of 35 s between the lowest and highest PI groups was assumed. Accordingly, taking 150 s as a representative onset time, the expected group means were set at 132.5, 150.0, and 167.5 s. A common standard deviation of 60 s was assumed to reflect the heterogeneity of real-world clinical data. Under these assumptions, with a two-sided alpha of 0.05 and 90% power, the required total sample size for a three-group comparison was estimated to be 227 patients.

### 2.9. Statistical Analysis

Statistical analyses were performed using R version 4.4.0 (R Foundation for Statistical Computing, Vienna, Austria). Categorical variables, including sex, ASA-PS class, anesthetic method, presence of hypertension, and presence of diabetes mellitus, are presented as numbers and percentages. The normality of continuous variables was assessed using the Shapiro–Wilk test. All continuous variables are presented as median [interquartile range], as none satisfied the normality assumption, and non-parametric methods were used for all group comparisons. Between-group comparisons of onset time across baseline PI groups were performed in the overall cohort and separately within each anesthetic subgroup (TIVA and volatile anesthesia) using the Kruskal–Wallis test. Post hoc pairwise comparisons used Dunn’s test with Bonferroni correction. Two-group comparisons used the Mann–Whitney U test. Univariable associations between continuous and binary categorical variables and onset time were assessed by Spearman rank correlation. For multivariable analysis, sequential (nested) multivariable linear regression model on log-transformed onset time was performed using sequential covariate blocks, with model fit assessed by adjusted R^2^. Baseline PI was entered as a continuous variable in the regression model, whereas group comparisons used the categorical PI classification (low-PI, intermediate-PI, and normal-PI). Post hoc power analysis was performed for the ΔPI_60_ tertile comparison using the observed effect size and sample size, confirming greater than 99.9% power. Although the a priori sample size calculation was performed for the primary outcome, the final analytic sample (n = 1377) substantially exceeds the minimum requirement and provides adequate statistical power for the multivariable regression analysis. A two-sided *p* < 0.05 was considered statistically significant with Bonferroni correction applied for multiple comparisons.

## 3. Results

### 3.1. Study Population and Baseline Characteristics

**Figure 1:**
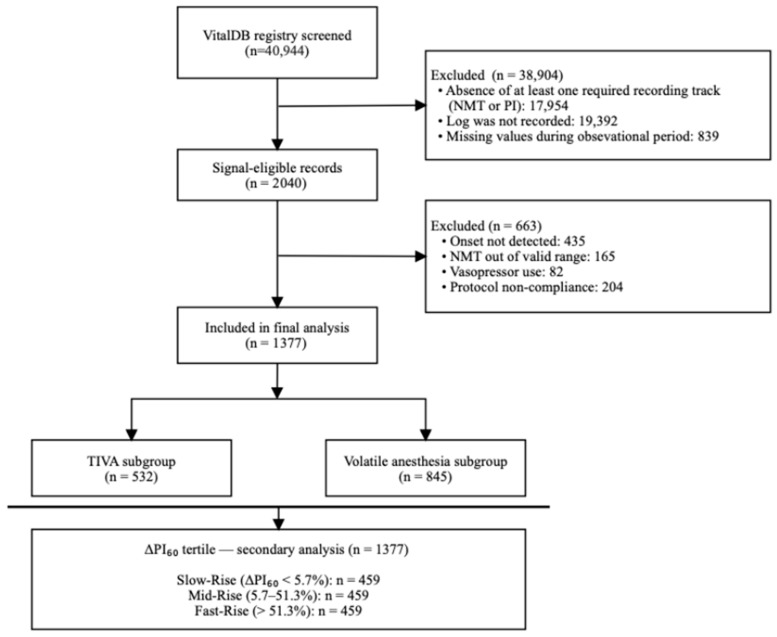
Patient selection flowchart. Of 40,944 patients screened from the institutional VitalDB registry, 38,904 were excluded due to absent recording tracks (NMT or PI), missing log data, or missing values during the observational period. The remaining 2040 signal-eligible records were further screened, with 663 excluded due to undetectable rocuronium onset, NMT out of valid range, vasopressor use during induction, or protocol non-compliance. The final cohort of 1377 patients was stratified by anesthetic method (TIVA, n = 532; volatile anesthesia, n = 845) for the primary analysis and by ΔPI_60_ tertile for the secondary analysis. NMT, neuromuscular transmission; PI, perfusion index; TIVA, total intravenous anesthesia; ΔPI_60_, relative change in perfusion index from immediately after propofol bolus to 60 s after propofol bolus.

Data from 40,944 patients in the institutional VitalDB registry were screened; 38,904 were excluded due to absent recording tracks, missing log data, or missing values during the observational period, yielding 2040 signal-eligible records. A further 663 were excluded due to undetectable onset, NMT out of valid range, vasopressor use during induction, or protocol non-compliance, leaving 1377 patients for final analysis: 532 in the TIVA group and 845 in the volatile anesthesia group (Figure 1, Table 1). Several baseline characteristics differed significantly across PI groups within each anesthetic subgroup, including female sex and baseline HR; these variables were included as covariates in the multivariable regression model. Baseline HR tended to be lower in the normal-PI group in both subgroups. Other variables showing subgroup-specific differences—hypertension and ΔMBP in the TIVA subgroup, and height and baseline MBP in the volatile subgroup—were either already adjusted for in the regression model or showed no significant correlation with onset time (Spearman ρ < 0.03), and are therefore unlikely to have materially confounded the primary outcome comparison.

### 3.2. Primary Outcome: Onset Time by Baseline PI Group

**Figure 2:**
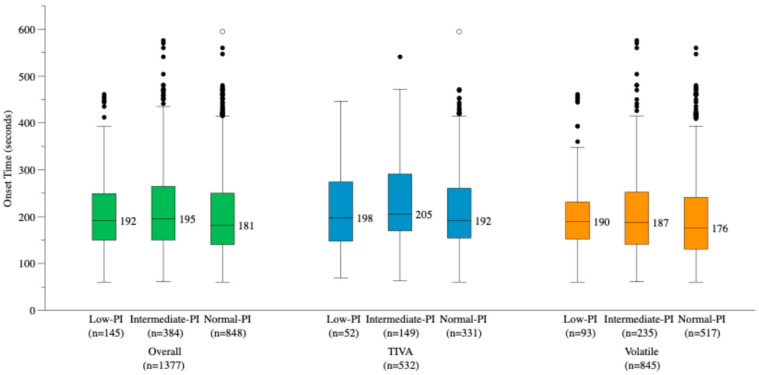
Rocuronium onset time by baseline perfusion index (PI) group and anesthetic method. Boxplots display the median and interquartile range whiskers represent 1.5 × interquartile range, and dots indicate outliers. In the overall cohort, onset time differed significantly across baseline PI groups (*p* = 0.033), though this was not replicated within either anesthetic subgroup (TIVA: *p* = 0.200; volatile: *p* = 0.137). TIVA, total intravenous anesthesia.

In the overall cohort, rocuronium onset time differed significantly across baseline PI groups (*p* = 0.033). Post hoc analysis with Bonferroni correction identified a significant difference between the intermediate-PI and normal-PI groups (*p* = 0.028); comparisons involving the low-PI group did not reach significance after correction. Within-subgroup comparisons did not reach statistical significance in either the TIVA subgroup (*p* = 0.200) or the volatile anesthesia subgroup (*p* = 0.137). Across all PI groups, volatile anesthesia was associated with significantly shorter onset time than TIVA (*p* < 0.001). Results are presented in Table 1 and Figure 2.

### 3.3. Selection of the Optimal ΔPI Time Point

To identify the time point at which ΔPI best predicted onset time, Spearman rank correlation and AUC were evaluated at t15, t30, t45, and t60. The correlation with onset time increased monotonically with time: ρ = −0.193 at t15, −0.244 at t30, −0.260 at t45, and −0.285 at t60 (all *p* < 0.001). AUC followed the same pattern: 0.582, 0.609, 0.617, and 0.633, respectively. No reverse causation was observed (zero patients had onset time < 60 s). Accordingly, t60 was selected as the primary ΔPI time point, and all subsequent analyses use ΔPI_60_. The median ΔPI_60_ in the overall cohort was 23.8% [0.0–72.2%]. Equal-frequency tertile cutoffs were 5.7% (33rd percentile) and 51.3% (67th percentile), yielding three groups of 459 patients each.

### 3.4. Secondary Outcome: Onset Time by ΔPI_60_ Tertile

**Figure 3:**
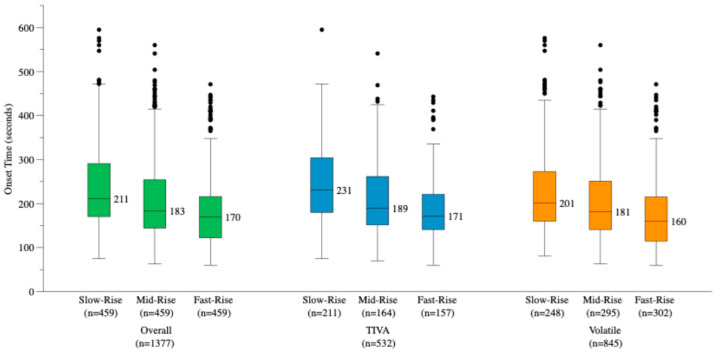
Rocuronium onset time by ΔPI_60_ tertile in the overall cohort and by anesthetic method subgroup. Boxplots display the median and interquartile range, whiskers represent 1.5 × interquartile range, and dots indicate outliers Numbers above each box indicate the median onset time in seconds. Patients were stratified into three equal-frequency tertiles: Slow-Rise (ΔPI_60_ < 5.7%), Mid-Rise (5.7% ≤ ΔPI_60_ ≤ 51.3%), and Fast-Rise (ΔPI_60_ > 51.3%). Onset time differed significantly across tertiles in the overall cohort (*p* < 0.001), and the effect was consistent in both the TIVA (*p* < 0.001) and volatile anesthesia subgroups (*p* < 0.001). All pairwise comparisons were significant after Bonferroni correction (all *p* < 0.001). ΔPI_60_, relative change in perfusion index from immediately after propofol injection to 60 s, expressed as a percentage. PI, perfusion index; TIVA, total intravenous anesthesia.

Onset time differed significantly across ΔPI_60_ tertiles (*p* < 0.001; post hoc power > 99.9%). Median onset times were 211 [170–291] s in the Slow-Rise group, 183 [144–254] s in the Mid-Rise group, and 170 [122–216] s in the Fast-Rise group, representing a 41 s difference between extreme tertiles (Figure 3). All pairwise post hoc comparisons were statistically significant after Bonferroni correction (all *p* < 0.001). The ΔPI_60_ tertile effect was consistent in both the TIVA subgroup (*p* < 0.001) and the volatile subgroup (*p* < 0.001). The Slow-Rise group showed a significantly greater rise in HR (*p* < 0.001) and a smaller fall in blood pressure (during induction compared with the Mid-Rise and Fast-Rise groups (Table 2), suggesting differential hemodynamic responses to induction across ΔPI_60_ tertiles.

### 3.5. Univariable Correlations with Onset Time and Multivariable Analysis

Among all candidate variables, ΔPI_60_ (%) showed the strongest inverse correlation with onset time (Spearman ρ = −0.285, *p* < 0.001) (Table 3). ΔHR (T0 to TOF0) showed the strongest positive correlation (ρ = +0.262, *p* < 0.001), indicating that a larger heart rate increase during induction was associated with longer onset.

**Figure 4:**
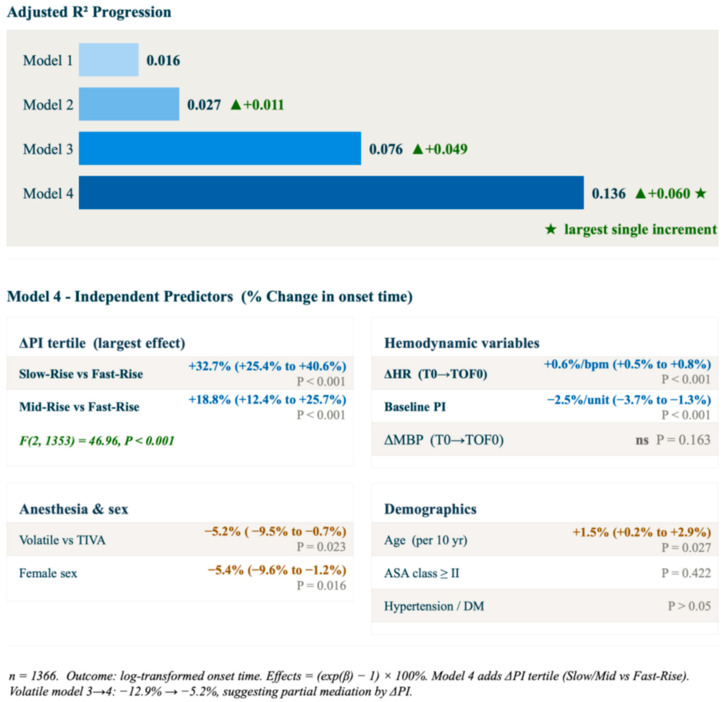
Nested ordinary least-squares regression on log-transformed rocuronium onset time. The upper panel shows the progression of adjusted R^2^ across four sequential models: Model 1 (demographics: sex, age, BMI, ASA class, hypertension, diabetes mellitus), Model 2 (+anesthetic method + baseline PI), Model 3 (+ ΔHR and ΔMBP from baseline to TOF0), and Model 4 (+ΔPI_60_ tertile). The lower panel presents the independent predictors in Model 4, expressed as percentage change in onset time [=(exp(β) − 1) × 100%] with 95% confidence intervals in parentheses. ΔPI_60_ tertile (Slow-Rise and Mid-Rise vs. Fast-Rise as reference) contributed the largest single increment in model fit (Δadj-R^2^ = 0.060; F(2, 1353) = 46.96, *p* < 0.001). The attenuation of the volatile anesthesia effect from Model 3 (−12.9%) to Model 4 (−5.2%) is consistent with partial mediation through ΔPI_60_. ★, largest single R^2^ increment; ns, not significant. ΔPI_60_, relative change in perfusion index from immediately after propofol bolus to 60 s after propofol bolus; ΔHR, heart rate change from baseline to TOF0; ΔMBP, mean blood pressure change from baseline to TOF0; TOF0, train-of-four count = 0; PI, perfusion index; TIVA, total intravenous anesthesia; BMI, body mass index; adj-R^2^, adjusted R-squared.

Results of the regression are presented in Figure 4. Model 1 (demographics only) explained 1.6% of variance (adjusted R^2^ = 0.016). Model 2 added anesthetic method and baseline PI (adjusted R^2^ = 0.035), and Model 3 further added ΔHR and ΔMBP (adjusted R^2^ = 0.076). The addition of ΔPI_60_ tertile in Model 4 produced the largest single increment (adjusted R^2^ = 0.136, increment Δ = 0.060 over Model 3; F(2, 1353) = 46.96, *p* < 0.001). In Model 4, the Slow-Rise and Mid-Rise tertiles were associated with 32.7% and 18.8% longer onset relative to the Fast-Rise reference, respectively (both *p* < 0.001). Female sex (−5.4%, *p* = 0.016), volatile anesthesia (−5.2%, *p* = 0.023), and baseline PI (−2.5% per unit, *p* < 0.001) also remained independently significant. The attenuation of the volatile anesthesia effect from Model 3 to Model 4 (−12.9% → −5.2%) is consistent with partial mediation through ΔPI_60_.

## 4. Discussion

This single-center retrospective cohort study demonstrated that ΔPI_60_ was most strongly associated with rocuronium onset time among all variables examined. This finding suggests that the early peripheral vasodilatory response during induction, captured non-invasively by pulse oximetry-derived PI, reflects the efficiency of rocuronium delivery to the NMJ and may serve as a physiologically grounded, bedside-accessible index of induction-phase perfusion dynamics.

Although we hypothesized that higher baseline PI would reflect superior peripheral perfusion and thereby predict faster rocuronium onset, baseline PI showed only a weak and inconsistent association with onset time (overall *p* = 0.033, ε^2^ = 0.004), and this overall significance was not replicated within either anesthetic subgroup (TIVA: *p* = 0.200; volatile: *p* = 0.137). This pattern of overall significance without within-subgroup replication is consistent with a confounding structure in which anesthetic method independently influences both baseline PI distribution and onset time, rather than reflecting a genuine direct relationship between baseline perfusion and rocuronium delivery. Indeed, the effect size was negligible (ε^2^ = 0.004), and the only significant post hoc comparison was between the intermediate-PI and normal-PI groups (*p* = 0.028), a finding that does not follow the monotonic dose-response gradient that would be expected if baseline perfusion were a direct pharmacodynamic determinant. Taken together, these findings suggest that baseline PI, as measured before induction, does not constitute a robust independent predictor of rocuronium onset time, and its apparent overall association should be interpreted with caution. The limited predictive value of baseline PI likely reflects the fact that baseline PI, measured before induction, is rapidly rendered less informative once anesthesia begins. Propofol induces peripheral vasodilation primarily through inhibition of sympathetic vasoconstrictor nerve activity, causing a rapid decrease in systemic vascular resistance within 60–90 s of bolus administration [14,15]. Concurrently, remifentanil reduces cardiac output predominantly via bradycardia and direct vasodilation [15]. The combined hemodynamic perturbation imposed by these two agents during induction substantially alters the peripheral perfusion state from baseline, rendering pre-induction PI an insufficient predictor of the perfusion conditions governing rocuronium delivery to the NMJ. Therefore, the key factor may not be the baseline perfusion but the dynamic changes in cardiac output and peripheral vascular resistance during induction. We therefore reasoned that monitoring the change in PI during induction would more directly capture the perfusion conditions relevant to rocuronium delivery.

PI is well positioned to reflect this dynamic process. PI integrates both stroke volume and peripheral vascular tone at the fingertip [8], placing it closer to the peripheral drug delivery step than conventional vital signs, such as HR or MBP. Notably, ΔPI during general anesthesia showed tight correlation with changes in cardiac output (r = 0.9) and stroke volume (r = 0.9) [16], supporting PI as a dynamic hemodynamic surrogate in the perioperative setting. These findings suggest that a rising PI after induction may reflect maintained or augmented cardiac output and peripheral perfusion, which conditions potentially favoring rocuronium delivery to the neuromuscular junction.

The observed association between ΔPI_60_ and rocuronium onset time in this study is physiologically consistent with the known hemodynamic effects of propofol-based induction. Propofol induces peripheral vasodilation with systemic vascular resistance reaching its nadir within 60–90 s of bolus administration [14]. This effect may be directly reflected in the photoplethysmographic signal and produces a durable increase in the pulsatile component of the photoplethysmographic waveform, which is the physiological basis of rising PI [17]. A larger ΔPI_60_ may therefore reflect a more favorable induction-phase hemodynamic response in which cardiac output and peripheral perfusion are relatively preserved, potentially supporting more efficient rocuronium delivery to the neuromuscular junction. Conversely, a flat or declining ΔPI_60_ may indicate sustained peripheral vasoconstriction or reduced stroke volume, conditions that could limit drug distribution at the capillary level and contribute to prolonged onset.

Based on these considerations, we propose the following mechanistic hypothesis. The degree of propofol-mediated sympatholysis shows substantial interindividual variability [18]. In patients in whom sympatholysis is incomplete, peripheral vascular resistance may be maintained despite induction, attenuating the expected rise in PI. Propofol’s early parasympatholytic effect may simultaneously shift sympathovagal balance toward relative sympathetic dominance, producing compensatory tachycardia [17]. This high-resistance, low-stroke-volume hemodynamic state would constrain capillary perfusion at the NMJ and contribute to prolonged rocuronium onset, consistent with the Slow-Rise pattern observed in this study. Several findings in the present study are consistent with this hypothesis. First, ΔHR showed a strong positive correlation with onset time (ρ = +0.262, *p* < 0.001), consistent with a compensatory tachycardia pattern in patients with reduced stroke volume and maintained peripheral vascular resistance. Second, the Slow-Rise group showed a significantly smaller fall in blood pressure (ΔMBP −4.0 vs. −13.0 mmHg, *p* = 0.017) and a significantly greater rise in heart rate (ΔHR +2.0 vs. −1.0 bpm, *p* < 0.001) during induction compared with the Mid-Rise and Fast-Rise groups, despite comparable baseline HR and MBP (both *p* > 0.05), indicating that these differences reflect induction-phase dynamics rather than pre-existing patient characteristics. Third, the ΔPI_60_ tertile comparison demonstrated the largest single increment in multivariable model fit (adjusted R^2^ Δ0.060, F(2, 1353) = 46.96, *p* < 0.001), indicating that the induction-phase PI response captures hemodynamic variance not explained by baseline characteristics, anesthetic method, or conventional vital sign changes alone. However, this interpretation remains speculative as cardiac output and stroke volume were not directly measured in this study.

The regression model provides further insight into the relative contributions of hemodynamic and pharmacodynamic factors to rocuronium onset variability. Model 1, comprising demographic variables alone, explained only 1.6% of variance (adjusted R^2^ = 0.016). The addition of anesthetic method and baseline PI in Model 2, followed by ΔHR and ΔMBP in Model 3, incrementally improved model fit to adjusted R^2^ = 0.076, reflecting the contribution of both the choice of anesthetic agent and induction-phase hemodynamic responses. The entry of ΔPI_60_ tertile in Model 4 produced the largest single increment (Δ0.060), increasing the explained variance by 79% over Model 3. This finding indicates that ΔPI_60_ captures a distinct aspect of induction phase physiology by reflecting dynamic peripheral perfusion, independent of baseline PI, anesthetic method, HR change, or blood pressure change. Nevertheless, the total explained variance of the final model remained modest (adjusted R^2^ = 0.136), and the discriminative ability of ΔPI_60_ was moderate (AUC = 0.633), indicating that induction-phase PI dynamics should be regarded as a physiologically plausible contributor to rocuronium onset variability rather than a clinically actionable standalone predictor.

The independent associations of older age (ρ = +0.093, *p* < 0.001), female sex (−5.4%, *p* = 0.016), and volatile anesthesia (−5.2%, *p* = 0.023) with rocuronium onset time in the multivariable model are consistent with prior pharmacodynamic literature. The prolongation of onset with increasing age likely reflects age-related reductions in cardiac output and muscle blood flow, which slow drug delivery to the NMJ [19]. The faster onset in female patients is consistent with the well-established finding that women require approximately 30% less rocuronium dose than men to achieve equivalent NMB, attributed to lower lean body mass per unit body weight [20]. The faster onset observed in the volatile anesthesia subgroup may be partly attributable to the direct potentiation of neuromuscular blockade by volatile agents at the nicotinic acetylcholine receptor [21], as well as their peripheral vasodilatory effects augmenting induction-phase perfusion.

This study has several limitations. First, the retrospective single-center design precludes causal inference and may introduce selection bias; furthermore, minor temporal inaccuracies in event logging cannot be entirely excluded. Additionally, non-invasive blood pressure was measured at 3 min intervals using a cuff placed on the same arm as NMT monitoring. Although cuff inflation is transient, intermittent reduction in forearm perfusion during cuff inflation may have transiently limited rocuronium delivery to the adductor pollicis, potentially affecting onset time measurement in a subset of patients. However, given the fixed 3 min measurement interval applied uniformly across all patients, any such effect is likely to have been minimal and distributed randomly rather than systematically, and is therefore unlikely to have materially biased the between-group comparisons. Second, cardiac output and systemic vascular resistance were not directly measured. Therefore, the mechanistic pathway from ΔPI_60_ to rocuronium delivery remains speculative, and the present findings should be considered hypothesis-generating rather than mechanistically conclusive. Third, PI was measured at the fingertip of one hand while NMT monitoring was performed on the contralateral adductor pollicis, and perfusion conditions at the two sites may not have been identical. Fourth, the overall variance explained (adjusted R^2^ = 0.136) remains modest, indicating that unmeasured biological and technical factors account for most of the interindividual variability in onset time. Fifth, the cohort was drawn from a single tertiary hospital with predominantly ASA-PS I–II patients, limiting external generalizability to higher-risk populations with significant autonomic or cardiovascular comorbidities. Sixth, although core temperature was measured and showed no significant difference across ΔPI_60_ tertile groups, PI at the fingertip is physiologically sensitive to peripheral temperature. We did not record peripheral skin temperature, and interindividual differences in cutaneous vascular tone may have contributed to ΔPI_60_ variability independently of systemic hemodynamic changes. Seventh, several baseline characteristics, including female sex and baseline heart rate, differed significantly across PI groups within each anesthetic subgroup. Variables with subgroup-specific differences showed no meaningful correlation with onset time (Spearman ρ < 0.03), and consistently differing variables were included as covariates in the multivariable regression model, supporting the robustness of the findings of the current study. Finally, several potential confounding factors were not accounted for in this analysis. Intravenous fluid administration during the induction period was not recorded and may have influenced both peripheral perfusion and rocuronium distribution. While propofol dosing was standardized by age-based protocol (2.0 mg/kg for patients aged <65 years and 1.5 mg/kg for patients ≥65 years), the speed of propofol injection was not controlled or recorded; variability in injection speed may have affected the rate of peripheral vasodilation and thus contributed to interindividual differences in ΔPI_60_ independently of the hemodynamic response. Furthermore, PI is derived from the pulsatile photoplethysmographic signal and may become unreliable or undetectable in patients with significant hemodynamic instability, severe peripheral vasoconstriction, or arrhythmia. The present findings should therefore not be extrapolated to extreme clinical scenarios in which PI signal integrity cannot be assured.

## 5. Conclusions

In conclusion, the early induction-phase change in peripheral perfusion index (ΔPI_60_) was most strongly associated with rocuronium onset time. These findings suggest that induction-phase peripheral perfusion dynamics, captured non-invasively by pulse oximetry-derived PI, represent a statistically significant and physiologically plausible contributor to rocuronium onset variability. Given the modest effect sizes and retrospective design, these findings should be regarded as hypothesis-generating. Prospective studies with direct hemodynamic measurements are needed to establish whether induction-phase PI dynamics may be useful for individualized NMB management.

## Figures and Tables

**Table 1:** Patients’ Characteristics.

	TIVA (n = 532)				Volatile Anesthesia (n = 845)			
Variable	Low-PI (n = 52)	Intermediate-PI (n = 149)	Normal-PI (n = 331)	*p* Value	Low-PI (n = 93)	Intermediate-PI (n = 235)	Normal-PI (n = 517)	*p* Value
Age (years)	54 [43–67]	60 [44–71]	59 [46–69]	0.460	59 [45–69]	61 [47–71]	61 [46–71]	0.570
Female sex, n (%)	24 (46.2)	80 (53.7)	135 (40.8)	0.031	50 (53.8)	126 (53.6)	227 (43.9)	0.022
Height (cm)	164.0 [158.4–171.7]	163.9 [157.7–169.5]	166.2 [158.9–171.6]	0.211	162.2 [157.5–168.9]	162.8 [156.6–169.2]	165.0 [158.0–171.9]	0.026
Weight (kg)	65.0 [56.6–71.2]	63.3 [55.7–71.1]	65.9 [57.5–73.0]	0.305	63.2 [55.4–70.0]	63.0 [53.8–69.8]	64.1 [56.8–72.0]	0.193
BMI (kg/m2)	23.4 [21.3–25.5]	23.4 [21.7–26.0]	23.9 [21.9–25.9]	0.541	23.4 [21.8–26.2]	23.6 [21.2–26.0]	23.8 [21.5–25.9]	0.956
ASA-PS I, n (%)	19 (36.5)	70 (47.0)	151 (45.6)	0.409	46 (49.5)	104 (44.3)	228 (44.1)	0.623
Hypertension, n (%)	23 (44.2)	41 (27.5)	126 (38.1)	0.033	27 (29.0)	89 (37.9)	197 (38.1)	0.237
Diabetes mellitus, n (%)	20 (38.5)	59 (39.6)	122 (36.9)	0.844	36 (38.7)	90 (38.3)	193 (37.3)	0.949
Body temperature (°C)	36.2 [35.6–36.8]	36.2 [35.4–36.7]	36.2 [35.5–36.5]	0.303	36.2 [35.2–36.6]	36.2 [35.5–36.6]	36.0 [35.2–36.5]	0.052
Baseline HR (bpm)	74 [64–85]	71 [63–84]	68 [60–77]	0.003	76 [69–89]	73 [66–85]	72 [64–80]	<0.001
ΔHR (bpm)	−0.5 [−8.0–11.8]	2.0 [−5.0–11.2]	1.5 [−4.0–12.0]	0.448	−0.5 [−8.2–7.0]	−0.5 [−8.0–7.0]	−1.0 [−7.0–6.0]	0.954
Baseline MBP (mmHg)	106 [96–115]	104 [94–115]	102 [92–114]	0.175	105 [96–116]	108 [96–120]	103 [92–116]	0.009
ΔMBP (mmHg)	−10.5 [−27.0–0.0]	−13.0 [−27.1–0.0]	−7.0 [−23.0–0.0]	0.030	−12.8 [−27.8–0.0]	−14.0 [−27.0–0.0]	−8.0 [−24.3–0.0]	0.120
Baseline PI	0.59 [0.46–0.66]	1.00 [0.87–1.20]	2.80 [1.80–4.10]	<0.001	0.55 [0.39–0.64]	1.00 [0.88–1.20]	3.00 [2.10–4.30]	<0.001
Onset time (s)	198 [149–270]	205 [170–291]	192 [154–260]	0.200	190 [152–231]	187 [141–252]	176 [131–241]	0.137

Data are expressed as number (%) or median [interquartile range]. ASA-PS, American Society of Anesthesiologists-Physical Status; BMI, body mass index.

**Table 2:** Baseline and induction-phase hemodynamic variables by ΔPI_60_ tertile.

Variable	Slow-Rise (n = 459)	Mid-Rise (n = 459)	Fast-Rise (n = 459)	*p* Value
Baseline HR (bpm)	70.0 [62.0–82.0]	72.0 [64.0–79.0]	72.0 [65.0–82.0]	0.063
Baseline MBP (mmHg)	103.0 [90.0–116.3]	104.0 [94.0–116.0]	105.0 [95.0–115.9]	0.104
ΔHR (bpm)	+2.0 [−4.0–+12.0]	−1.0 [−6.0–+6.0]	−1.0 [−8.0–+6.0]	<0.001
ΔMBP (mmHg)	−4.0 [−24.1–0.0]	−13.0 [−25.8–0.0]	−12.0 [−25.0–0.0]	0.017

Data are presented as median [interquartile range]. ΔHR, heart rate change from t0 to TOF0; ΔMBP, mean blood pressure change from t0 to TOF0. Tertile cutoffs: Slow-Rise (ΔPI_60_ < 5.7%), Mid-Rise (5.7% ≤ ΔPI_60_ ≤ 51.3%), Fast-Rise (ΔPI_60_ > 51.3%).

**Table 3:** Spearman Rank Correlations with Rocuronium Onset Time.

Variable	Spearman ρ	95% CI	*p* Value
**PI-related variables**			
ΔPI_60_ (%)	−0.285	−0.333, −0.236	<0.001
Baseline PI	−0.060	−0.113, −0.007	0.026
**Hemodynamic variable**			
ΔHR, T0 to TOF0 (bpm)	+0.262	+0.212, +0.311	<0.001
ΔMBP, T0 to TOF0 (mmHg)	−0.029	−0.082, +0.024	0.278
Pre-induction HR (bpm)	−0.095	−0.147, −0.042	<0.001
**Demographic variables**			
Age (years)	+0.093	+0.040, +0.145	<0.001
BMI (kg/m^2^)	+0.035	−0.018, +0.087	0.199
Body temperature (°C)	+0.004	−0.051, +0.058	0.889
**Categorical variables**			
Female sex	−0.101	−0.150, −0.048	<0.001
Hypertension	+0.028	−0.025, +0.080	0.300
Volatile vs. TIVA	−0.109	−0.158, −0.062	<0.001

ΔPI, relative change in perfusion index; ΔHR, heart rate change from baseline to TOF0; ΔMBP, mean blood pressure change from baseline to TOF0; BMI, body mass index; TIVA, total intravenous anesthesia; TOF0, train-of-four count = 0.

## Data Availability

The data presented in this study are available upon request from the corresponding author. The data are not publicly available due to privacy restrictions, as the Institutional Review Board approval did not include provisions for sharing raw patient data externally.

## Abbreviations

The following abbreviations are used in this manuscript:

ASA-PS	American Society of Anesthesiologists Physical Status
AUC	Area under the curve
BMI	Body mass index
Ce	Effect-site concentration
EMG	Electromyography
EMR	Electronic medical records
GCRP	Good Clinical Research Practice
HR	Heart rate
MBP	Mean blood pressure
NMB	Neuromuscular blockade
NMBA	Neuromuscular blocking agent
NMJ	Neuromuscular junction
NMT	Neuromuscular transmission
PI	Perfusion index
TCI	Target-controlled infusion
TIVA	Total intravenous anesthesia
TOF	Train-of-four
ΔHR	Heart rate change from baseline to train-of-four count = 0
ΔMBP	Mean blood pressure change from baseline to train-of-four count = 0
ΔPI_60_	Relative change in perfusion index from immediately after propofol bolus to 60 s after propofol bolus
